# Glycemic variability and the short-term mortality of hospitalized patients with COVID-19: a meta-analysis

**DOI:** 10.3389/fendo.2026.1846640

**Published:** 2026-06-16

**Authors:** Zixuan Wang, Jingyi Wang, Qiao Wu, Baozeng Wang

**Affiliations:** Department of Infectious Diseases, Beijing Tiantan Hospital, Capital Medical University, Beijing, China

**Keywords:** Coronavirus disease 2019, glucose fluctuation, glycemic variability, meta-analysis, mortality

## Abstract

**Background:**

Glucose variability (GV) reflects fluctuations in blood glucose and may better capture metabolic instability than static glycemic measures in patients with Coronavirus disease 2019 (COVID-19). However, its association with mortality remains uncertain due to heterogeneous study designs and inconsistent findings. This meta-analysis was performed to evaluate the association between GV and short-term all-cause mortality in adult patients hospitalized with COVID-19.

**Methods:**

PubMed, Embase, and Web of Science were systematically searched for longitudinal observational studies reporting the association between GV and mortality in patients hospitalized with COVID-19. Risk ratios (RRs) with 95% confidence intervals (CIs) were pooled using a random-effects model accounting for the possible influence of heterogeneity.

**Results:**

Ten cohort studies comprising 11 datasets and 77,395 patients were included, with 8,189 deaths. High GV was associated with a significantly increased risk of all-cause mortality (RR = 2.10, 95% CI: 1.69–2.59; I² = 45%). The association was stronger in studies with a mean age ≥ 62 years (RR = 2.79) compared with < 62 years (RR = 1.78; *p* for subgroup difference = 0.03). Results were consistent across GV metrics, analytic models, adjustment for diabetes status, and study quality (all p > 0.05). Meta-regression analysis showed that mean age demonstrated a borderline association with the effect estimate (coefficient = 0.020, *p* = 0.07), explaining a moderate proportion of heterogeneity (adjusted R² = 49.2%).

**Conclusions:**

Higher GV is associated with increased short-term mortality in hospitalized patients with COVID-19, supporting its role as a potential prognostic marker.

**Systematic Review Registration:**

https://www.crd.york.ac.uk/prospero/, identifier CRD420261358246.

## Introduction

Since its emergence in late 2019, Coronavirus disease 2019 (COVID-19), caused by SARS-CoV-2, has imposed a substantial global health burden (1, 2). Although most infected individuals experience mild illness, a considerable proportion of hospitalized patients develop severe complications, including acute respiratory failure, systemic inflammation, and multiorgan dysfunction, resulting in significant short-term mortality (3, 4). Reported mortality rates among hospitalized patients vary depending on disease severity, comorbidities, and healthcare capacity, but remain clinically important, particularly among older individuals and those with underlying conditions (5–7). Accordingly, early risk stratification has become a central component of clinical management. Established prognostic factors include advanced age, male sex, pre-existing comorbidities such as diabetes and cardiovascular disease, and laboratory markers reflecting inflammation, coagulation abnormalities, and organ dysfunction (8, 9). However, these conventional indicators may not fully capture the dynamic metabolic disturbances that occur during acute illness, underscoring the need to identify additional prognostic markers.

Among these, disturbances in glucose homeostasis have attracted increasing attention (10). Beyond chronic hyperglycemia, acute glycemic dysregulation during hospitalization has been associated with adverse outcomes in patients with COVID-19, irrespective of pre-existing diabetes status (11, 12). Glucose variability (GV), defined as fluctuations in blood glucose levels over time during the acute phase of illness (13), may provide incremental prognostic information beyond static glycemic measures (14). Mechanistically, increased GV may promote oxidative stress, endothelial dysfunction, and inflammatory activation, thereby contributing to disease progression and organ injury (15, 16). However, current evidence regarding the association between GV and mortality in COVID-19 remains inconsistent (17–26), largely due to heterogeneity in study design, patient populations, and GV assessment methods. Therefore, this meta-analysis was conducted to systematically synthesize the available evidence and clarify the relationship between GV and short-term mortality in hospitalized patients with COVID-19.

## Methods

The meta-analysis was carried out in accordance with established methodological guidance, following the principles outlined in the PRISMA 2020 statement (27) and the Cochrane Handbook for Systematic Reviews and Meta-Analyses (28), encompassing protocol planning, study selection, data collection, statistical analysis, and results interpretation. The study protocol was registered prospectively in the PROSPERO database (registration number: CRD420261358246).

### Database search

A systematic literature search was conducted in PubMed, Embase, and Web of Science to identify studies that met the eligibility criteria for inclusion. The search strategy was constructed using the combination of the following terms: (1) “glycemic variability” OR “glycemic fluctuation” OR “glucose variability” OR “glucose fluctuation” OR “standard deviation of blood glucose” OR “coefficient of variation of blood glucose” OR “glycemic lability index” OR “GLI” OR “mean amplitude of glycemic excursion” OR “MAGE” OR “largest amplitude of glycemic excursion” OR “LAGE”; (2) “coronavirus” OR “severe acute respiratory syndrome coronavirus 2” OR “SARS-CoV-2” OR “novel coronavirus” OR “nCoV” OR “2019-nCoV” OR “COVID-19” OR “COVID”; and (3) “mortality” OR “death” OR “survival” OR “deaths” OR “prognosis” OR “outcome” OR “prospective” OR “prospectively” OR “retrospective” OR “retrospectively” OR “followed” OR “follow-up” OR “longitudinal” OR “cohort”. Only full-text, peer-reviewed articles published in English and involving human participants were eligible for inclusion. Additionally, the reference lists of relevant reviews and original studies were manually examined to identify further potentially eligible publications. All databases were searched from their inception up to February 18, 2026. Detailed search strategies for each database are presented in **Supplementary File 1**.

### Study inclusion and exclusion criteria

The selection of studies was guided by the PICOS principle:

P (Population): Adult patients (≥ 18 years) hospitalized with confirmed COVID-19.

I (Exposure): High GV assessed during hospitalization, defined using metrics such as standard deviation of blood glucose (SDBG), coefficient of variation of blood glucose (CVBG), mean amplitude of glycemic excursions (MAGE), ranges between the highest and the lowest blood glucose, or other parameters in the original studies. The cutoffs for defining a high GV were also consistent with the values used in the original studies.

C (Comparator): Low GV, defined according to study-specific thresholds or categorizations.

Outcome (O): All-cause mortality during hospitalization or within 30 days, compared between patients with the lowest vs. the highest category of GV. In studies reporting 28-day mortality, this outcome was considered equivalent to 30-day mortality for the purpose of this meta-analysis, given their clinical comparability in acute illness settings.

S (Study design): Observational studies (prospective or retrospective cohort, nested case–control studies) with longitudinal follow-up, or *post-hoc* analysis of randomized controlled trials.

Studies were excluded if they met any of the following criteria: (1) not limited to patients with COVID-19; (2) included pediatric populations or non-hospitalized patients; (3) did not evaluate GV as an exposure or failed to provide a clear definition or quantification of GV; (4) did not report all-cause mortality or lacked sufficient data to estimate the association between GV and mortality risk; (5) were cross-sectional studies, case reports, case series, reviews, editorials, letters, or preclinical studies; or (6) involved overlapping populations, in which case the one with the largest sample size was included.

### Study quality assessment

Two reviewers independently performed the literature search, screened studies for eligibility, extracted data, and assessed study quality. Any disagreements were resolved through discussion, and when necessary, a third investigator was consulted to reach consensus. The methodological quality of the included studies was evaluated using the Newcastle–Ottawa Scale (NOS) (29), which assesses study quality across three domains: selection, comparability, and outcome assessment. NOS scores range from 1 to 9, with studies scoring ≥ 8 considered to be of high quality.

### Data collection

Data extraction was conducted independently by two reviewers using a standardized and pre-tested data collection form. Extracted variables included study characteristics (first author, publication year, country, and study design), patient characteristics (diagnosis, sample size, mean age, sex distribution, and proportions of patients with diabetes), exposure details (metrics used for evaluating GV, methods for determining the cutoff values for defining high GV, and duration for GV evaluation), follow-up duration, number of patients who died during follow-up, and variables adjusted for in multivariable analyses examining the association between GV and mortality risk of patients with COVID-19.

### Statistical analyses

The association between GV and all-cause mortality of hospitalized patients with COVID-19 was evaluated by pooling risk ratios (RRs) with their corresponding 95% confidence intervals (CIs) (28), compared between patients with high vs. low GV. When necessary, effect estimates and their standard errors were derived from the reported 95% CIs or *p* values. Before pooling, all effect measures were converted to the natural logarithmic scale to improve normality and ensure variance stabilization for meta-analysis (28). To evaluate variability across studies, we applied the Cochrane Q test and calculated the I² statistic (30). Heterogeneity was categorized based on I² values as low (< 25%), moderate (25–75%), or high (> 75%). Pooled effect estimates were calculated using the inverse variance (IV) approach within a random-effects framework (DerSimonian–Laird method) to account for potential between-study variability (28). Sensitivity analyses were performed using a leave-one-out approach, in which each study was sequentially excluded to assess the stability and robustness of the pooled results (31). To identify possible sources of between-study variability, we performed predefined subgroup analyses stratified by mean age of the patients, proportion of men, metrics for evaluating GV, analytic model (univariate vs. multivariate), adjustment of the diabetic status of the patients (yes vs. no), and NOS scores. Median values of study-level continuous variables (e.g., mean age) were used as cutoffs to define subgroups. This approach was applied to ensure a balanced distribution of studies between subgroups and to improve the stability and interpretability of subgroup comparisons. Additionally, univariate meta-regression analyses were conducted to examine the potential impact of study-level characteristics on the association between GV and mortality risk (28). The variables assessed included mean age, proportion of male patients, proportion of patients preexisting diabetes, and study quality scores via NOS. Publication bias was assessed through visual inspection of funnel plot symmetry and further evaluated using Egger’s regression test (32). A two-sided *p* value < 0.05 was considered indicative of statistical significance. All analyses were performed using RevMan (version 5.3; Cochrane Collaboration, Oxford, UK) and Stata (version 17.0; StataCorp, College Station, TX, USA).

## Results

### Database search results

**Figure 1:**
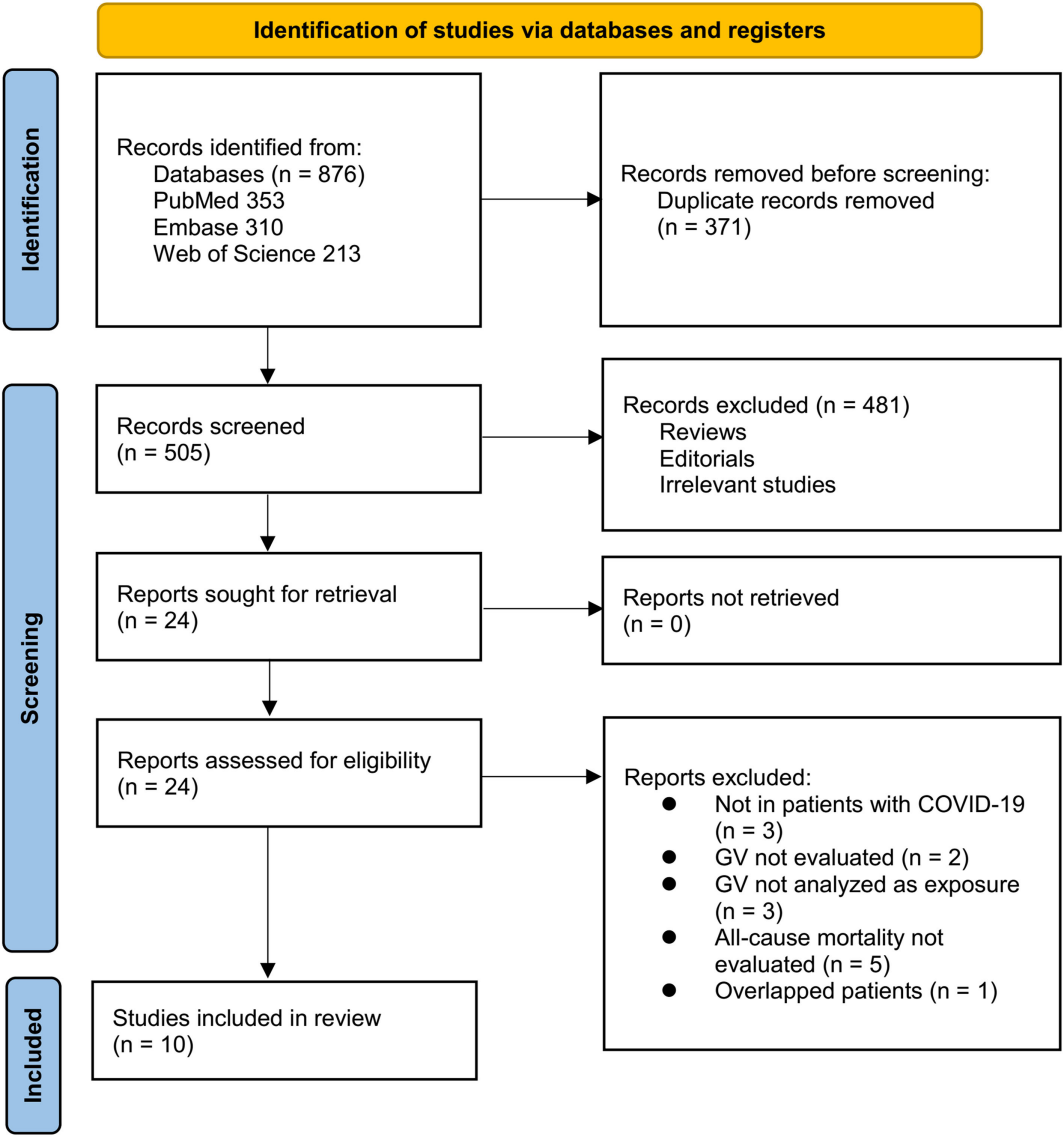
Flow diagram of the study selection process.

The study selection process is illustrated in **Figure 1**. A total of 876 records were retrieved from the three databases, of which 371 duplicates were removed. Following title and abstract screening, 481 records were excluded for not meeting the predefined inclusion criteria. The full texts of 24 articles were subsequently evaluated independently by two reviewers, and 14 were excluded for the reasons detailed in **Figure 1**. Ultimately, ten studies met the eligibility criteria and were included in the quantitative meta-analysis (17–26).

### Overview of study characteristics

The main characteristics of the included studies are summarized in **Table 1** (17–26). A total of 10 cohort studies, comprising 11 independent datasets, published between 2020 and 2024 were included in this meta-analysis, involving 77,395 hospitalized patients with COVID-19. One study (Abuhasira et al., 2022) (22) reported results separately for patients with and without diabetes mellitus, and these were treated as independent datasets in the analysis. The included studies consisted of one prospective (25) and nine retrospective (17–24, 26) cohort studies. The studies were conducted across multiple regions, including China, Israel, USA, Germany, and Brazil, indicating broad geographic diversity. The mean age of patients ranged from 57.0 to 71.0 years, and the proportion of men ranged from 45.6% to 67.9%, reflecting a predominantly middle-aged to elderly population with a moderate male predominance. In all included studies, GV was calculated based on intermittent glucose measurements obtained during routine clinical care. None used continuous glucose monitoring (CGM), and detailed measurement modalities (e.g., capillary vs. venous sampling) were not consistently reported. GV was assessed using diverse indices, including standard deviation of blood glucose (SDBG) (19, 21, 25), coefficient of variation (CVBG) (22, 26), glucose range (17, 18, 20, 24), and derived measures such as daily variability of glucose (DVG) (23). The duration of GV assessment ranged from the first day of ICU admission to the entire hospitalization period or predefined time windows (e.g., first 7–10 days). Definitions of high GV varied across studies, including absolute thresholds, quartile-based comparisons (e.g., Q4 vs Q1), and composite dysglycemia definitions. Mortality was primarily assessed as in-hospital (17, 19–26) or 28-day mortality (18). In total, 8,189 deaths were reported across the included datasets. Seven studies (17–19, 21, 22, 25, 26) provided adjusted effect estimates controlling for key confounders such as age, sex, comorbidities, disease severity, and baseline glucose levels to a varying extent, although three studies reported only unadjusted results (20, 23, 24).

**Table 1:** Characteristics of the included studies.

Study	Country	Design	Diagnosis	Sample size	Mean age (years)	Men (%)	DM (%)	Index for GV	Duration of GV evaluation	Cutoff of high GV	Follow-up duration (days)	Number of patients died	Variables adjusted
Zhu (18)	China	RC	Confirmed COVID-19 with preexisting T2D	810	63.3	53.6	100	Range between lowest FBG and highest 2hPG	During hospitalization	10 mmol/L	28	62	Age, sex, indicators of severity of COVID-19, comorbidities (hypertension, CHD, cerebrovascular diseases, chronic liver diseases, and chronic renal diseases), and hospital site
Rao (17)	USA	RC	Confirmed COVID-19 (PCR-positive)	63	62.1	55.6	NR	Range between lowest and highest BG	First 7 days of hospitalization	Median (5.8 mmol/L)	In-hospital	16	Age, sex, and diabetic status
Chen (19)	China	RC	Confirmed COVID-19 (PCR-positive)	548	57	45.6	18.1	SDBG	First 7 days of hospitalization	Q4:Q1	In-hospital	59	Age, sex, comorbidities, glucocorticoid use, baseline CURB-65 score, and baseline inflammation markers (CRP, PCT, D-dimer, LDH), and baseline glucose level
Xie (21)	China	RC	Confirmed COVID-19 (PCR-positive)	101	61	49.5	14.9	SDBG	During hospitalization (blood glucose monitoring at 8 time points daily)	2.0 mmol/L (previous study defined)	In-hospital	16	Age, sex, and severity of COVID-19 at admission
Morse (20)	USA	RC	Confirmed COVID-19 (PCR-positive) inpatients	74,148	62.8	52.9	36.5	Presence of both hypoglycemia (< 3.9 mmol/L) and hyperglycemia (> 10 mmol/L) during hospitalization	During hospitalization	Hypoglycemia (< 3.9 mmol/L) and hyperglycemia (> 10 mmol/L)	In-hospital	7536	None
Abuhasira (22) NDM	Israel	RC	Confirmed COVID-19 (PCR-positive) without DM	314	65.1	62.7	0	CVBG	During hospitalization	Q4:Q1	In-hospital	38	Age, sex, BMI, hypertension, dyslipidemia, AF, last values of WBC, D-dimer, ferritin, and CRP
Abuhasira (22) DM	Israel	RC	Confirmed COVID-19 (PCR-positive) with DM	251	71	61.8	100	CVBG	During hospitalization	Q4:Q1	In-hospital	80	Age, sex, BMI, hypertension, dyslipidemia, AF, last values of WBC, D-dimer, ferritin, and CRP
Hartmann (23)	Germany	RC	Confirmed COVID-19 with ARDS	106	63	67.9	30.2	DVG, median of absolute differences between successive daily FBG levels	During hospitalization	1.4 mmol/L (previous study defined)	In-hospital	53	None
Parolin (25)	Brazil	PC	Confirmed COVID-19 (RT-PCR positive)	628	59.6	65.8	32.8	SDBG	During hospitalization (first 10 days for patients with longer stays)	2.5 mmol/L (ROC curve analysis derived)	In-hospital	131	Age, sex, and comorbidities (hypertension, DM, CVD, cerebrovascular disease, chronic kidney injury, dyslipidemia, malignancy, obesity, pulmonary disease), and use of dexamethasone during hospitalization
Bellaver (24)	Brazil	RC	Confirmed SARS-CoV-2 infection with ICU admission	187	60	60	42.7	Range between lowest and highest BG	First day of ICU admission	2.2 mmol/L (previous study defined)	In-hospital	80	None
Boschi (26)	Brazil	RC	Confirmed COVID-19 (RT-PCR or antigen)	239	60	56.1	27.6	CVBG	During the first 10 days of ICU hospitalization	Q4:Q1	In-hospital	118	Age, sex, SAPS-3 score, SOFA score, obesity, number of comorbidities, and DM

In Morse 2021, GV was defined as a composite binary variable based on the presence of both hypoglycemia (< 3.9 mmol/L) and hyperglycemia (> 10 mmol/L) during hospitalization, rather than a continuous or variability-based metric.

NR, not reported; RC, retrospective cohort; PC, prospective cohort; COVID-19, coronavirus disease 2019; PCR, polymerase chain reaction; RT-PCR, reverse transcription polymerase chain reaction; ICU, intensive care unit; DM, diabetes mellitus; T2D, type 2 diabetes; GV, glucose variability; BG, blood glucose; FBG, fasting blood glucose; 2hPG, 2-hour postprandial glucose; SDBG, standard deviation of blood glucose; CVBG, coefficient of variation of blood glucose; DVG, daily variability of glucose; ARDS, acute respiratory distress syndrome; Q1, first quartile; Q4, fourth quartile; BMI, body mass index; CHD, coronary heart disease; AF, atrial fibrillation; WBC, white blood cell; CRP, C-reactive protein; PCT, procalcitonin; LDH, lactate dehydrogenase; CVD, cardiovascular disease; SAPS-3, Simplified Acute Physiology Score 3; SOFA, Sequential Organ Failure Assessment; ROC, receiver operating characteristic; NDM, non-DM.

### Study quality evaluation

The methodological quality of the included studies was assessed using the NOS, with detailed results presented in **Table 2**. The NOS scores ranged from 7 to 9, indicating overall moderate to high methodological quality. Five studies achieved the maximum score of 9, reflecting strong cohort representativeness, appropriate selection of comparison groups, adequate exposure ascertainment, comprehensive control for confounding factors, and reliable outcome assessment (17, 21, 22, 25, 26). Two datasets scored 8, mainly due to limited representativeness of the exposed cohort (18, 19). The remaining four datasets scored 7, primarily owing to insufficient adjustment for confounding variables despite adequate outcome assessment and follow-up (20, 23, 24). Overall, the included datasets were considered to be of moderate to high quality, supporting the robustness of the pooled estimates while recognizing some methodological heterogeneity.

**Table 2:** Study quality evaluation via the Newcastle-Ottawa scale.

Study	Representativeness of the exposed cohort	Selection of the non-exposed cohort	Ascertainment of exposure	Outcome not present at baseline	Control for age	Control for other confounding factors	Assessment of outcome	Enough long follow-up duration	Adequacy of follow-up of cohorts	Total
Zhu (18)	0	1	1	1	1	1	1	1	1	8
Rao (17)	1	1	1	1	1	1	1	1	1	9
Chen (19)	0	1	1	1	1	1	1	1	1	8
Xie (21)	1	1	1	1	1	1	1	1	1	9
Morse (20)	1	1	1	1	0	0	1	1	1	7
Abuhasira (22)	1	1	1	1	1	1	1	1	1	9
Hartmann (23)	1	1	1	1	0	0	1	1	1	7
Parolin (25)	1	1	1	1	1	1	1	1	1	9
Bellaver (24)	1	1	1	1	0	0	1	1	1	7
Boschi (26)	1	1	1	1	1	1	1	1	1	9

### Results of the meta-analysis

**Figure 2:**
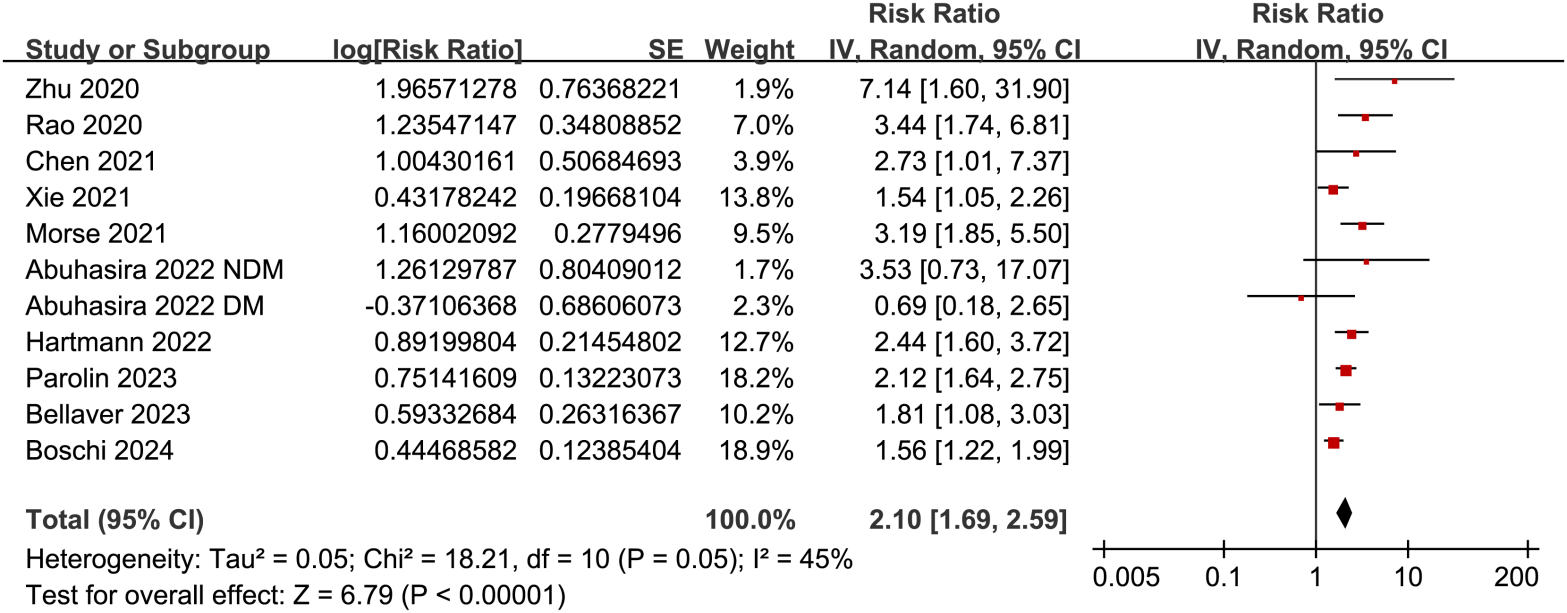
Forest plots showing the meta-analysis of the association between GV and short-term mortality of hospitalized patients with COVID-19.

The pooled analysis of the included studies (17–26) demonstrated that high GV during hospitalization was associated with an increased risk of all-cause mortality in hospitalized patients with COVID-19 (RR: 2.10, 95% CI: 1.69 to 2.59, *p* < 0.001; **Figure 2**) with moderate heterogeneity (Cochrane Q test *p* = 0.05; I² = 45%). Leave-one-out sensitivity analyses yielded consistent results, with pooled RRs ranging from 1.99 to 2.23 (all *p* < 0.05), indicating the robustness of the overall estimate.

### Results of the subgroup analysis

**Figure 3:**
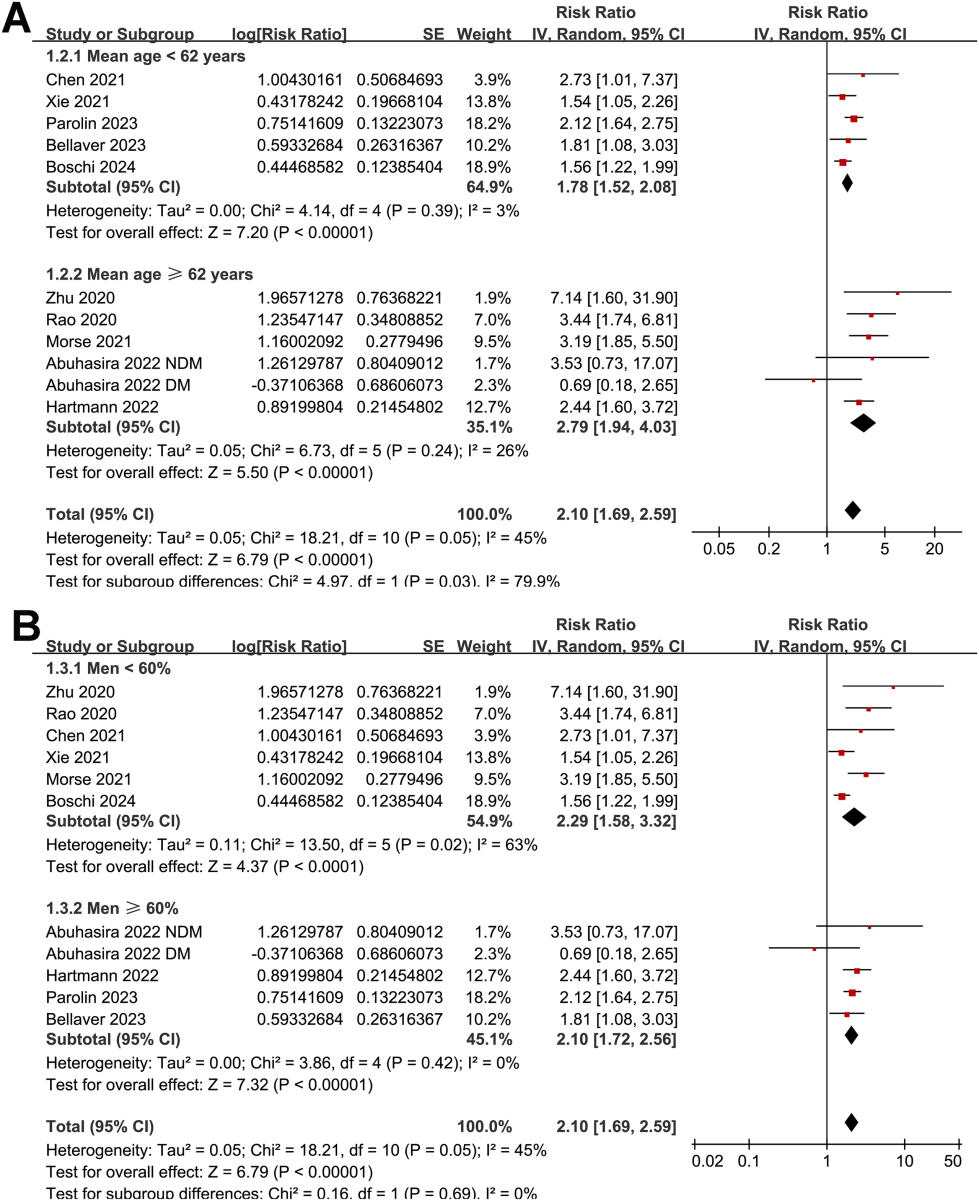
Forest plots showing the subgroup analysis of the association between GV and short-term mortality of hospitalized patients with COVID-19: **(A)** forest plots for the subgroup analysis according to the mean age of the patients; and **(B)** forest plots for the subgroup analysis according to the proportion of men.

**Figure 4:**
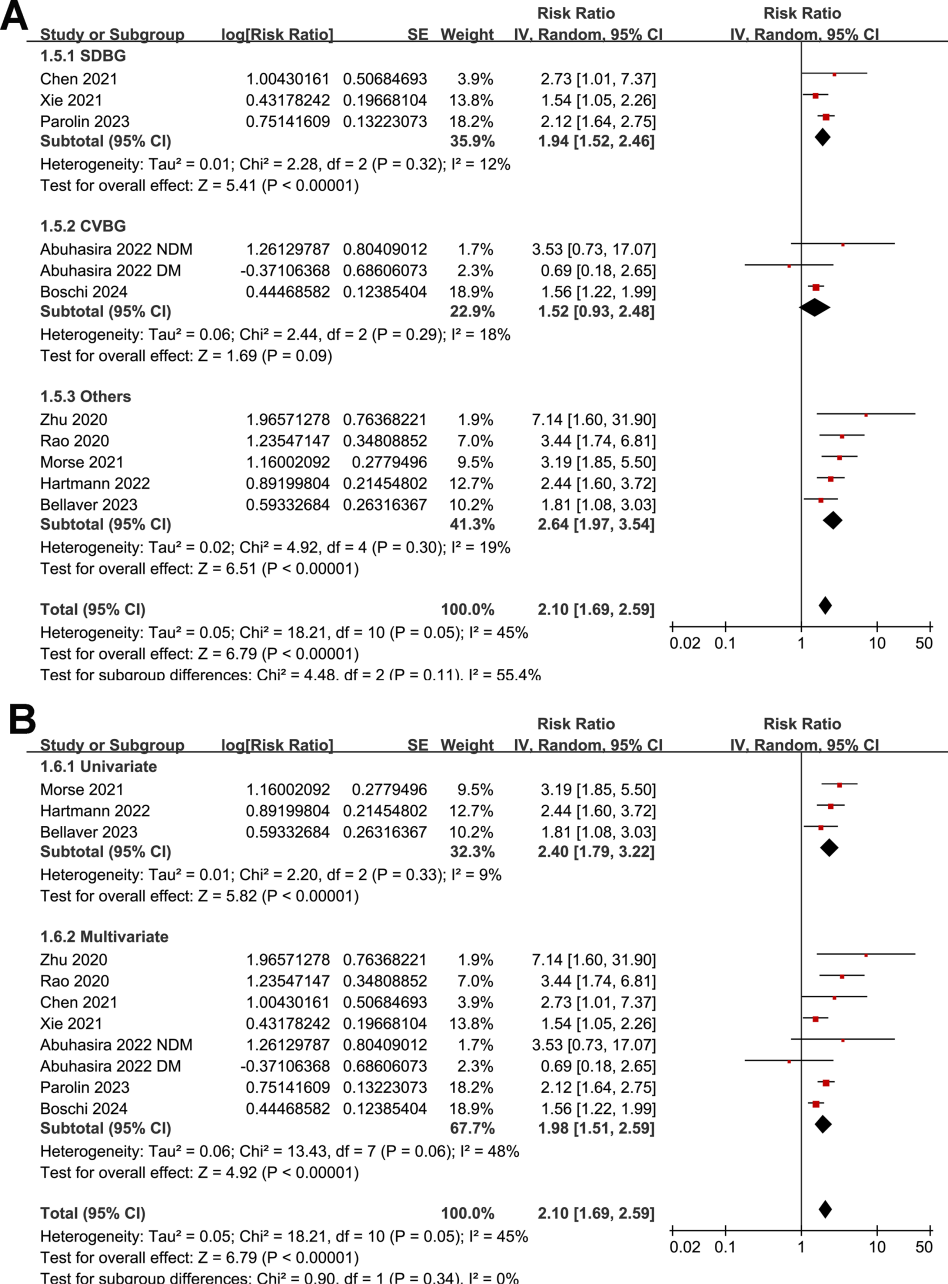
Forest plots showing the subgroup analysis of the association between GV and short-term mortality of hospitalized patients with COVID-19: **(A)** forest plots for the subgroup analysis according to the parameters of GV; and **(B)** forest plots for the subgroup analysis according to the analytic models.

**Figure 5:**
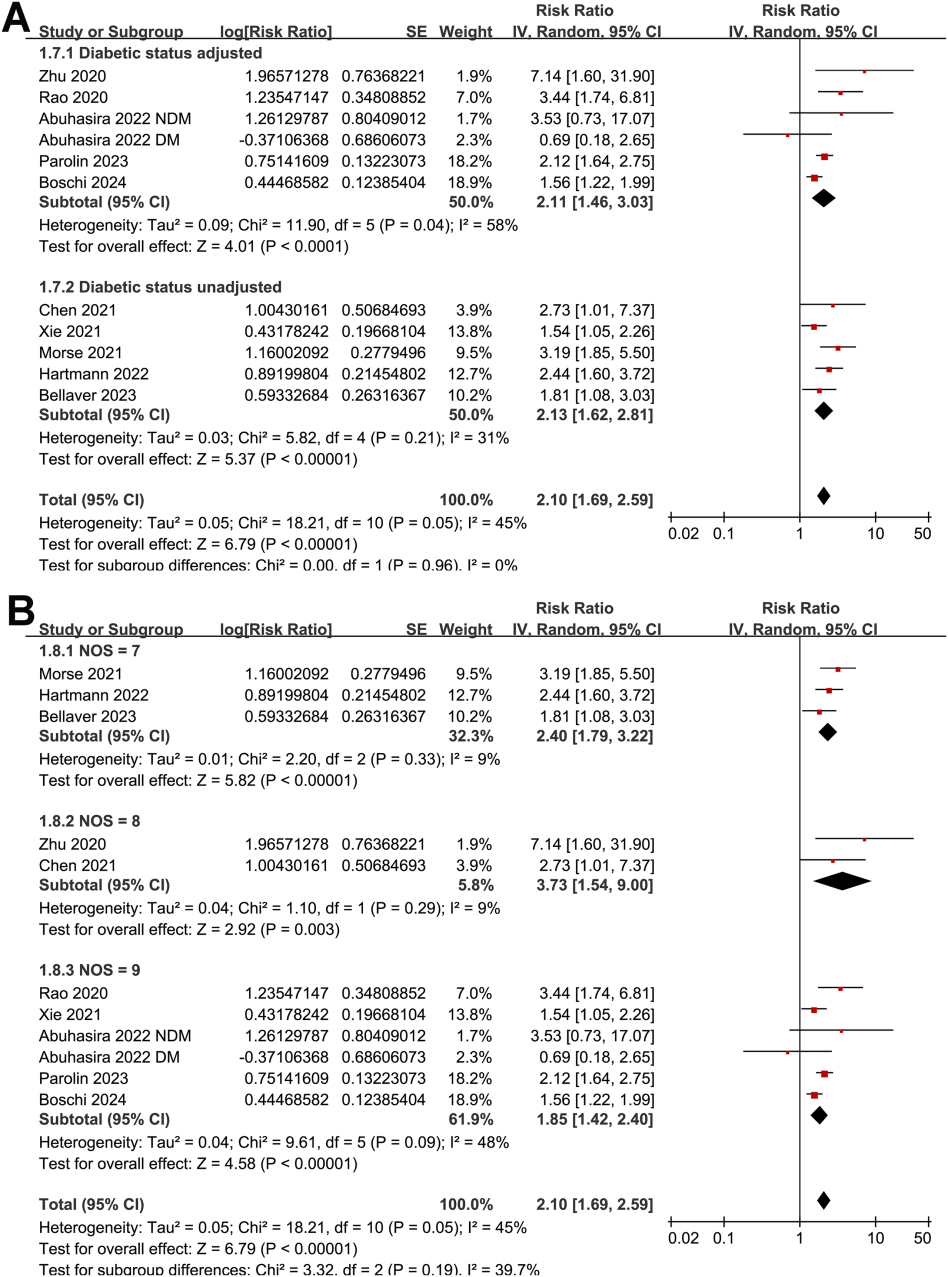
Forest plots showing the subgroup analysis of the association between GV and short-term mortality of hospitalized patients with COVID-19: **(A)** forest plots for the subgroup analysis according to the adjustment of the diabetic status of the patients; and **(B)** forest plots for the subgroup analysis according to the NOS scores.

Subgroup analyses suggested a stronger association between high GV and mortality risk in patients with COVID-19 with the mean age ≥ 62 years compared to those < 62 years (RR: 2.79 vs. 1.78, *p* for subgroup difference = 0.03; **Figure 3A**). The association was similar between studies with a proportion of men < 60% or ≥ 60% (RR: 2.29 vs. 2.10, *p* for subgroup difference = 0.69; **Figure 3B**). In addition, the results were consistent for studies evaluating GV with SDBG, CVBG, and other metrics (RR: 1.94, 1.52, and 2.64, *p* for subgroup difference = 0.11; **Figure 4A**), and between studies with the univariate and multivariate analyses (RR: 2.40 vs. 1.98, *p* for subgroup difference = 0.34; **Figure 4B**). No significant differences were observed between studies with or without the adjustment of the diabetic status (RR: 2.11 vs. 2.13; *p* for subgroup difference = 0.96; **Figure 5A**), or between studies with NOS scores of 7, 8, and 9 (RR: 2.40, 3.73, and 1.85, *p* for subgroup difference = 0.19; **Figure 5B**).

### Results of the meta-regression analysis

Univariate meta-regression analysis was performed to explore potential sources of heterogeneity, as summarized in **Table 3**. The results showed that mean age demonstrated a borderline association with the effect estimate (coefficient = 0.020, *p* = 0.07), explaining a moderate proportion of heterogeneity (adjusted R² = 49.2%). In contrast, the proportion of men, prevalence of diabetes, and study quality assessed by the NOS score were not significantly associated with the effect size (all *p* > 0.05), although diabetes prevalence and NOS score accounted for modest proportions of heterogeneity (adjusted R² = 29.8% and 39.9%, respectively).

**Table 3:** Results of univariate meta-regression analysis.

Variables	RR for the association between GV and short-term mortality of patients with COVID-19			
	Coefficient	95% CI	*p* values	Adjusted R^2^
Mean age (years)	0.020	-0.002 to 0.042	0.07	49.2%
Men (%)	0.00068	-0.04139 to 0.04275	0.97	0%
DM (%)	0.0049	-0.0101 to 0.0199	0.48	29.8%
NOS	-0.15	-0.40 to 0.11	0.23	39.9%

RR, risk ratio; CI, confidence interval; GV, glycemic variability; DM, diabetes mellitus; COVID-19, coronavirus disease 2019; NOS, Newcastle-Ottawa Scale.

### Publication bias

**Figure 6:**
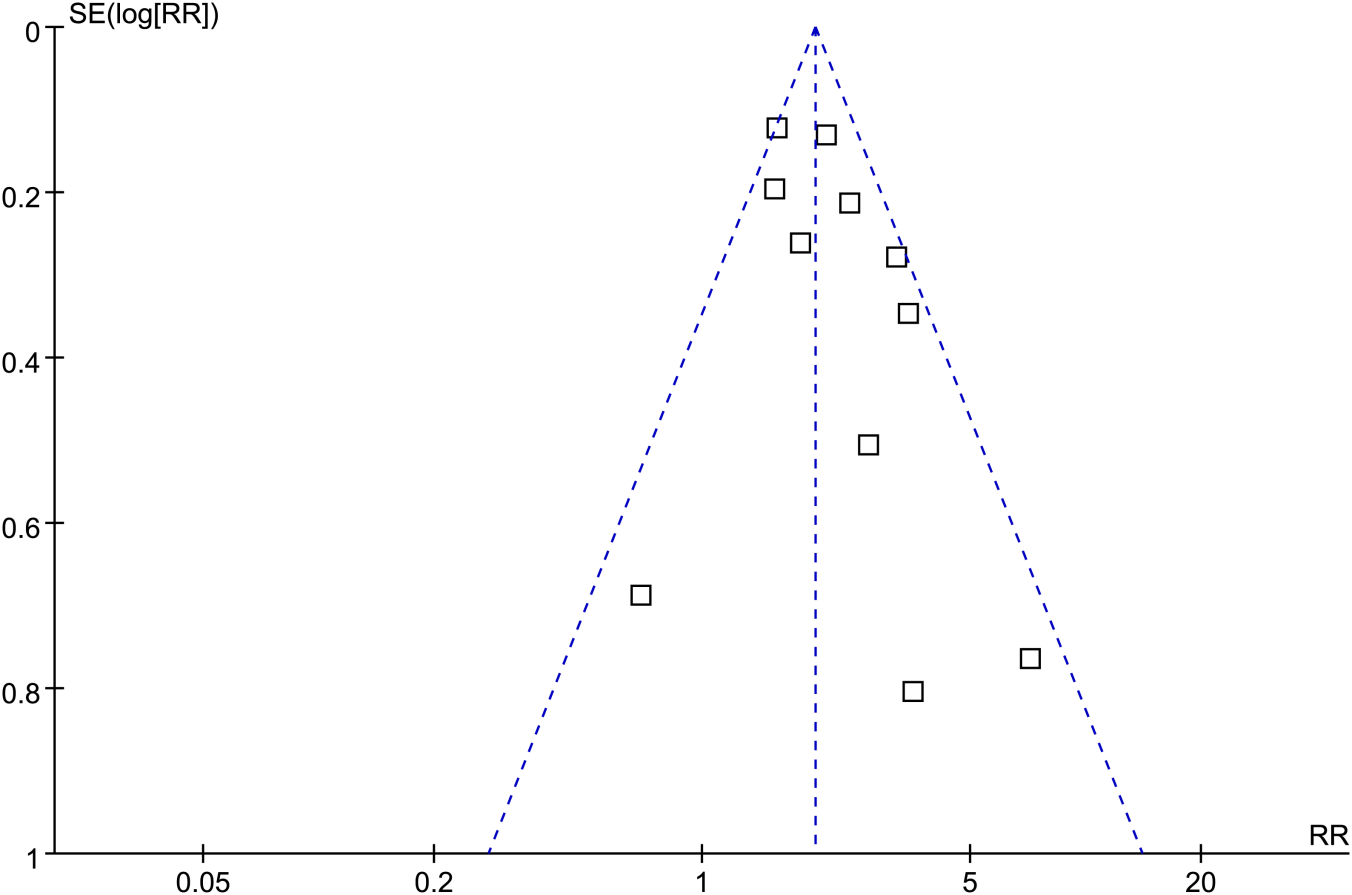
Funnel plots evaluating the publication bias for the meta-analysis of the association between GV and short-term mortality of hospitalized patients with COVID-19.

As illustrated in **Figure 6**, the funnel plots assessing the association between GV and mortality risk of hospitalized patients with COVID-19 appeared generally symmetrical. In line with this observation, Egger’s regression test did not detect significant publication bias (*p* = 0.79). Nevertheless, these findings should be interpreted with caution due to the relatively small number of included studies (k = 10).

## Discussion

In this meta-analysis of 10 cohort studies comprising 11 datasets, we demonstrated that higher GV during hospitalization is associated with an increased risk of short-term mortality in patients with COVID-19. Importantly, this association was consistent across different GV metrics, analytical models, and adjustment strategies, suggesting that GV may represent a robust prognostic marker beyond conventional glycemic indicators. Rather than merely reflecting chronic glycemic status, GV captures acute metabolic instability during critical illness (33), which may better reflect the dynamic physiological perturbations in patients with COVID-19. These findings extend existing evidence linking dysglycemia to adverse outcomes and support the concept that not only absolute glucose levels but also their fluctuations may carry important prognostic information.

Several biological mechanisms may explain the association between increased GV and poor prognosis in COVID-19. Fluctuating glucose levels have been shown to induce greater oxidative stress and endothelial dysfunction than sustained hyperglycemia (34, 35), leading to increased production of reactive oxygen species and impairment of vascular integrity (36). This may amplify the inflammatory response, which is already heightened in COVID-19, and contribute to cytokine release, endothelial permeability, and microvascular injury (37). In addition, GV has been associated with activation of pro-inflammatory pathways and dysregulation of immune responses, potentially impairing host defense against viral infection (38, 39). From a clinical perspective, higher GV often reflects a combination of factors, including disease severity, stress hyperglycemia, corticosteroid use, and variability in nutritional intake and insulin therapy (40, 41). These factors may collectively contribute to metabolic instability and organ dysfunction. These findings extend existing evidence linking dysglycemia to adverse outcomes in patients with COVID-19.

Beyond its association with mortality, accumulating evidence suggests that GV is linked to a range of adverse clinical outcomes in patients with COVID-19. Studies using CGM have shown that higher GV and greater time spent outside the target glucose range are associated with impaired respiratory function, increased risk of critical illness, and prolonged hospitalization (42, 43). For example, higher coefficients of variation and increased time above range have been correlated with worse oxygenation status and composite adverse outcomes, including intensive care unit admission and need for mechanical ventilation (44). Similarly, greater time above the target range has been linked to increased in-hospital complications, such as acute respiratory distress syndrome and acute kidney injury (45). These findings further support the concept that GV reflects underlying metabolic instability and may serve as a clinically relevant marker beyond static glycemic indices.

The subgroup analyses in this meta-analysis provide additional insights into the observed association. The stronger effect observed in studies with older populations suggests that aging-related vulnerability, including impaired metabolic flexibility and higher burden of comorbidities, may exacerbate the adverse impact of GV (46). In contrast, the lack of significant differences across GV metrics indicates that the association is not driven by a specific method of GV assessment, but rather reflects a general phenomenon of glycemic instability. Similarly, consistent results across studies with and without adjustment for diabetes status suggest that the prognostic significance of GV is not confined to patients with pre-existing diabetes, but may also apply to those with stress-induced dysglycemia. The meta-regression analysis further showed that mean age demonstrated a borderline association with the effect estimate, explaining a moderate proportion of heterogeneity, whereas other variables, including sex distribution, diabetes prevalence, and study quality, were not significant modifiers. These findings suggest that while patient characteristics such as age may contribute to between-study variability, the overall association between GV and mortality remains relatively stable.

This study has several strengths. First, we performed an up-to-date and comprehensive literature search across multiple databases, ensuring the inclusion of the most recent evidence. Second, all included studies were longitudinal cohort studies, allowing for a more reliable assessment of temporal relationships between GV and mortality. Third, we conducted multiple subgroup and meta-regression analyses to explore potential sources of heterogeneity and assess the robustness of the findings. In addition, sensitivity analyses confirmed the stability of the pooled results, further supporting the reliability of our conclusions.

However, several limitations should be acknowledged. Most included studies were retrospective in nature, which may introduce selection bias and information bias (47). The observational design also precludes causal inference, and residual confounding cannot be excluded, particularly given the variability in adjustment for clinical factors such as disease severity, treatment strategies, and glycemic management. There was also heterogeneity in the definitions and measurement of GV, including differences in metrics, monitoring frequency, and duration of assessment. Importantly, GV in all included studies was derived from intermittent glucose measurements rather than CGM, which may limit the precision and comparability of GV assessment across studies. Notably, one study (20) defined GV as a composite binary variable reflecting the coexistence of hypoglycemia and hyperglycemia, rather than using a continuous variability-based metric, which may have contributed to between-study heterogeneity. In addition, this study (20) contributed a substantially larger sample size than the others and reported unadjusted effect estimates with moderate methodological quality. Although this may raise concerns regarding potential bias or overrepresentation, sensitivity analyses demonstrated that exclusion of this study did not materially change the pooled estimate, suggesting that the overall findings were not driven by this dataset alone. Moreover, consistent results across subgroup analyses based on analytic models further support the robustness of the observed association. Furthermore, individual patient data (IPD) were not available, limiting our ability to perform more detailed analyses, such as dose–response relationships or standardized subgroup analyses. For example, subgroup analyses based on baseline glycemic control (e.g., HbA1c levels) or individual diabetes status were not feasible due to inconsistent reporting and the lack of IPD, which may limit more detailed exploration of effect modifiers. Besides, the observational design also precludes causal inference, and residual confounding cannot be excluded, particularly given the variability in adjustment for clinical factors such as disease severity, treatment strategies, and glycemic management. In addition, higher GV may partly reflect treatment-related factors, such as intensive insulin therapy or glucocorticoid use, which are themselves associated with disease severity and may influence mortality risk, introducing the possibility of indication bias. Publication bias cannot be entirely ruled out, although statistical testing did not suggest significant asymmetry. These limitations highlight the need for cautious interpretation of the findings.

From a clinical perspective, our results suggest that GV may serve as a useful marker for risk stratification in hospitalized patients with COVID-19. Monitoring and minimizing glucose fluctuations may represent a potential target for improving outcomes, although direct evidence supporting interventional strategies is currently lacking. Given the complex interplay between glycemic control, inflammation, and disease severity, future studies should investigate whether interventions aimed at reducing GV can improve clinical outcomes. Prospective studies with standardized GV assessment and comprehensive adjustment for confounders are warranted. In addition, randomized controlled trials evaluating glycemic management strategies, including the use of CGM and individualized insulin protocols, may help clarify whether targeting GV has a causal impact on prognosis.

## Conclusions

In conclusion, this meta-analysis demonstrates that higher GV is associated with increased short-term mortality in hospitalized patients with COVID-19. While the findings support the potential role of GV as a prognostic marker, they should be interpreted with caution due to the observational nature of the included studies. Further well-designed prospective and interventional studies are needed to determine whether reducing GV can improve clinical outcomes in this population.

## Data Availability

The original contributions presented in the study are included in the article/**Supplementary Material**. Further inquiries can be directed to the corresponding author.
