# Supplementary material for: Glycemic variability and the short-term mortality of hospitalized patients with COVID-19: a meta-analysis
Source: Front Endocrinol (Lausanne). 2026 Jun 16;17:1846640. doi: 10.3389/fendo.2026.1846640 (PMC13314431; doi:10.3389/fendo.2026.1846640)
Supplement: Supplementary file 1 [file DataSheet1.docx]

**Detailed search strategy for each database**

**PubMed**

(("Blood Glucose"[Mesh] OR "Glycemic Index"[Mesh] OR "Glucose Metabolism Disorders"[Mesh] OR "glycemic variability"[tiab] OR "glycemic fluctuation"[tiab] OR "glucose variability"[tiab] OR "glucose fluctuation"[tiab] OR "standard deviation of blood glucose"[tiab] OR "SDBG"[tiab] OR "coefficient of variation of blood glucose"[tiab] OR "CVBG"[tiab] OR "glycemic lability index"[tiab] OR "GLI"[tiab] OR "mean amplitude of glycemic excursion"[tiab] OR "MAGE"[tiab] OR "largest amplitude of glycemic excursion"[tiab] OR "LAGE"[tiab])) AND ("COVID-19"[Mesh] OR "SARS-CoV-2"[Mesh] OR "coronavirus"[tiab] OR "severe acute respiratory syndrome coronavirus 2"[tiab] OR "SARS-CoV-2"[tiab] OR "novel coronavirus"[tiab] OR "nCoV"[tiab] OR "2019-nCoV"[tiab] OR "COVID-19"[tiab] OR "COVID"[tiab]) AND ("Mortality"[Mesh] OR "Survival"[Mesh] OR "Prognosis"[Mesh] OR "mortality"[tiab] OR "death"[tiab] OR "deaths"[tiab] OR "survival"[tiab] OR "prognosis"[tiab] OR "outcome"[tiab] OR "prospective"[tiab] OR "prospectively"[tiab] OR "retrospective"[tiab] OR "retrospectively"[tiab] OR "followed"[tiab] OR "follow-up"[tiab] OR "longitudinal"[tiab] OR "cohort"[tiab])

**Embase**

('blood glucose'/exp OR 'glycemic control'/exp OR 'glucose variability'/exp OR 'glycemic variability':ti,ab OR 'glycemic fluctuation':ti,ab OR 'glucose variability':ti,ab OR 'glucose fluctuation':ti,ab OR 'standard deviation of blood glucose':ti,ab OR SDBG:ti,ab OR 'coefficient of variation of blood glucose':ti,ab OR CVBG:ti,ab OR 'glycemic lability index':ti,ab OR GLI:ti,ab OR 'mean amplitude of glycemic excursion':ti,ab OR MAGE:ti,ab OR 'largest amplitude of glycemic excursion':ti,ab OR LAGE:ti,ab) AND ('covid-19'/exp OR 'severe acute respiratory syndrome coronavirus 2'/exp OR coronavirus:ti,ab OR 'severe acute respiratory syndrome coronavirus 2':ti,ab OR 'SARS-CoV-2':ti,ab OR 'novel coronavirus':ti,ab OR nCoV:ti,ab OR '2019-nCoV':ti,ab OR 'COVID-19':ti,ab OR COVID:ti,ab) AND ('mortality'/exp OR 'survival'/exp OR 'prognosis'/exp OR mortality:ti,ab OR death:ti,ab OR deaths:ti,ab OR survival:ti,ab OR prognosis:ti,ab OR outcome:ti,ab OR prospective:ti,ab OR prospectively:ti,ab OR retrospective:ti,ab OR retrospectively:ti,ab OR followed:ti,ab OR 'follow-up':ti,ab OR longitudinal:ti,ab OR cohort:ti,ab)

**Web of Science**

TS = (("glycemic variability" OR "glycemic fluctuation" OR "glucose variability" OR "glucose fluctuation" OR "standard deviation of blood glucose" OR SDBG OR "coefficient of variation of blood glucose" OR CVBG OR "glycemic lability index" OR GLI OR "mean amplitude of glycemic excursion" OR MAGE OR "largest amplitude of glycemic excursion" OR LAGE)) AND TS = ("coronavirus" OR "severe acute respiratory syndrome coronavirus 2" OR "SARS-CoV-2" OR "novel coronavirus" OR "nCoV" OR "2019-nCoV" OR "COVID-19" OR "COVID") AND TS = ("mortality" OR "death" OR "deaths" OR "survival" OR "prognosis" OR "outcome" OR "prospective" OR "prospectively" OR "retrospective" OR "retrospectively" OR "followed" OR "follow-up" OR "longitudinal" OR "cohort")
